# Acetyl-CoA synthetase 2(ACSS2): a review with a focus on metabolism and tumor development

**DOI:** 10.1007/s12672-022-00521-1

**Published:** 2022-07-07

**Authors:** Rui Ling, Gong Chen, Xiang Tang, Na Liu, Yuepeng Zhou, Deyu Chen

**Affiliations:** 1 Institute of Oncology, Affiliated Hospital of Jiangsu University, Zhenjiang, China; 2 Department of Thoracic Surgery, Affiliated Hospital of Jiangsu University, Zhenjiang, China

**Keywords:** ACSS2, Acetyl-CoA, Metabolism, Acetylation modification

## Abstract

Acetyl-CoA synthetase 2 (ACSS2), an important member of the acetyl-CoA synthetase (ACSS) family, can catalyze the conversion of acetate to acetyl coenzyme A (acetyl-CoA). Currently, acetyl-CoA is considered an important intermediate metabolite in the metabolism of energy substrates. In addition, nutrients converge through acetyl-CoA into a common metabolic pathway, the tricarboxylic acid cycle and oxidative phosphorylation. Not only does ACSS2 play a crucial role in material energy metabolism, it is also involved in the regulation of various acetylation processes, such as regulation of histone and transcription factor acetylation. ACSS2-mediated regulation of acetylation is related to substance metabolism and tumorigenesis. In mammalian cells, ACSS2 utilizes intracellular acetate to synthesize acetyl-CoA, a step in the process of DNA and histone acetylation. In addition, studies in tumors have shown that cancer cells adapt to the growth conditions in the tumor microenvironment (TME) by activating or increasing the expression level of ACSS2 under metabolic stress. Therefore, this review mainly outlines the role of ACSS2 in substance metabolism and tumors and provides insights useful for investigating ACSS2 as a therapeutic target.

## Introduction

Metabolic reprogramming has been shown to be one of the indications of cancer [1]. In solid tumors, due to structural and functional microvascular abnormalities, cancer cells tend to grow rapidly and uncontrollably, a process that often results in a lack of nutrients and oxygen [2, 3]. To acquire sufficient energy and the cellular components essential for rapid proliferation, the metabolic program in tumor cells generally supports the increased production of protein, nucleic acid and lipid synthesis intermediates and thus maintain rapid proliferation [4]. In cancer, metabolic enhancement is the most dramatic change that increases glucose uptake and the utilization of aerobic glycolysis, a phenomenon also called the Warburg effect [5, 6]. Specifically in the presence of oxygen, cancer cells can reduce their energy metabolism by limiting glycolysis, thereby reprogramming glucose metabolism and energy production to enter a metabolic state referred to as aerobic glycolysis. In addition, cancer-related metabolic reprogramming can increase the biosynthesis of other macromolecules, such as proteins, nucleic acids, and lipids. The oxygenation status (both normoxia and hypoxia) in tumors does not remain constant but undergoes temporal and regional shifts [7], possibly because of the variability and dysregulation of tumor-associated neovascular organization.

**Fig. 1:**
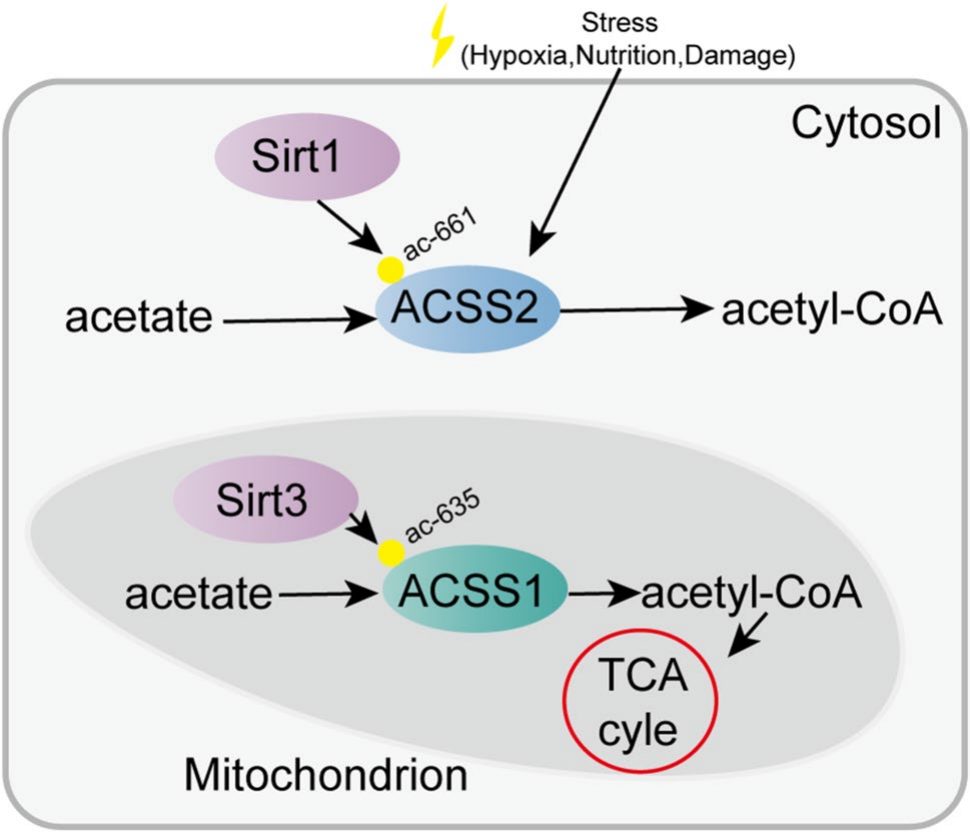
Difference between ACSS2 and ACSS1. Acetate is converted to acetyl-CoA by ACSS2 in the cytoplasm and ACSS1 in mitochondria. SIRT1 activates ACSS2 through lysine 661 deacetylation, while SIRT3 activates ACSS1 in the mitochondrial matrix through lysine 635 deacetylation. ACSS2 is correlated with nutrient deprivation, hypoxia, injury, etc.

Acetyl-CoA synthetase 2 (ACSS2) is a crucial member of the acetyl-CoA synthetase (ACSS) family and is transcribed from the ACSS2 gene. The ACSS2 gene, first isolated from yeast cells, encodes two isoforms produced by alternative transcription start site selection, with lengths of 1606 bp and 2106 bp [8]. The ACSS enzyme is the sole known mammalian enzyme that can catalyze the conversion of free acetate into acetyl coenzyme A (acetyl-CoA). The three known isoforms of human ACSS are termed ACSS1, ACSS2, and ACSS3 [8, 9]. To date, the enzymatic activity and function of ACSS3 are still insufficiently understood. In HepG2 hepatoma cells, ACSS3 was found to have no effect on lipid synthesis or histone acetylation [10]. The main substrate of ACSS1 and ACSS2 is acetate, while the preferential substrate of ACSS3 is propionate [11]. Both acetylation and deacetylation of specific lysine sites modulate the enzymatic activity of ACSS1, ACSS2, and ACSS3. Both ACSS1 and ACSS2 are inhibited by acetylation but reactivated by deacetylation. Sirtuin 1 (SIRT1) activates ACSS2 in the nucleus and cytoplasm through deacetylation of lysine 661, while Sirtuin 3 (SIRT3) activates ACSS1 in the mitochondrial matrix through deacetylation of lysine 635 (Fig. 1) [12]. Two acetate related enzymes, ACSS1 and ACSS2, differ in their tissue distribution and subcellular localization [8, 13]. On the one hand, as a mitochondrial matrix enzyme, ACSS1 is expressed mainly in cardiac and skeletal muscle as well as brown adipose tissue. On the other hand, as a nuclear and cytoplasmic enzyme, ACSS2 is strongly expressed in the liver, kidney and heart and moderately expressed in the brain and testis, as shown by early studies [14]. ACSS2 participates in lipid synthesis and facilitates protein acetylation by generating acetyl-CoA, while ACSS1 is involved in acetate oxidation [9]. The functional differences in these enzymes involve energy production through the tricarboxylic acid (TCA) cycle. Due to its more thorough utilization of intracellular acetate, ACSS2 is expressed in almost all cell types under different physiological conditions [15]. In addition, ACSS2 expression is highly correlated with nutrient deprivation, hypoxia, injury, immune activation, etc. [16–18]. This article mainly introduces the role of ACSS2 in substance metabolism and tumors.

## ACSS2 and acetyl-CoA

**Fig. 2:**
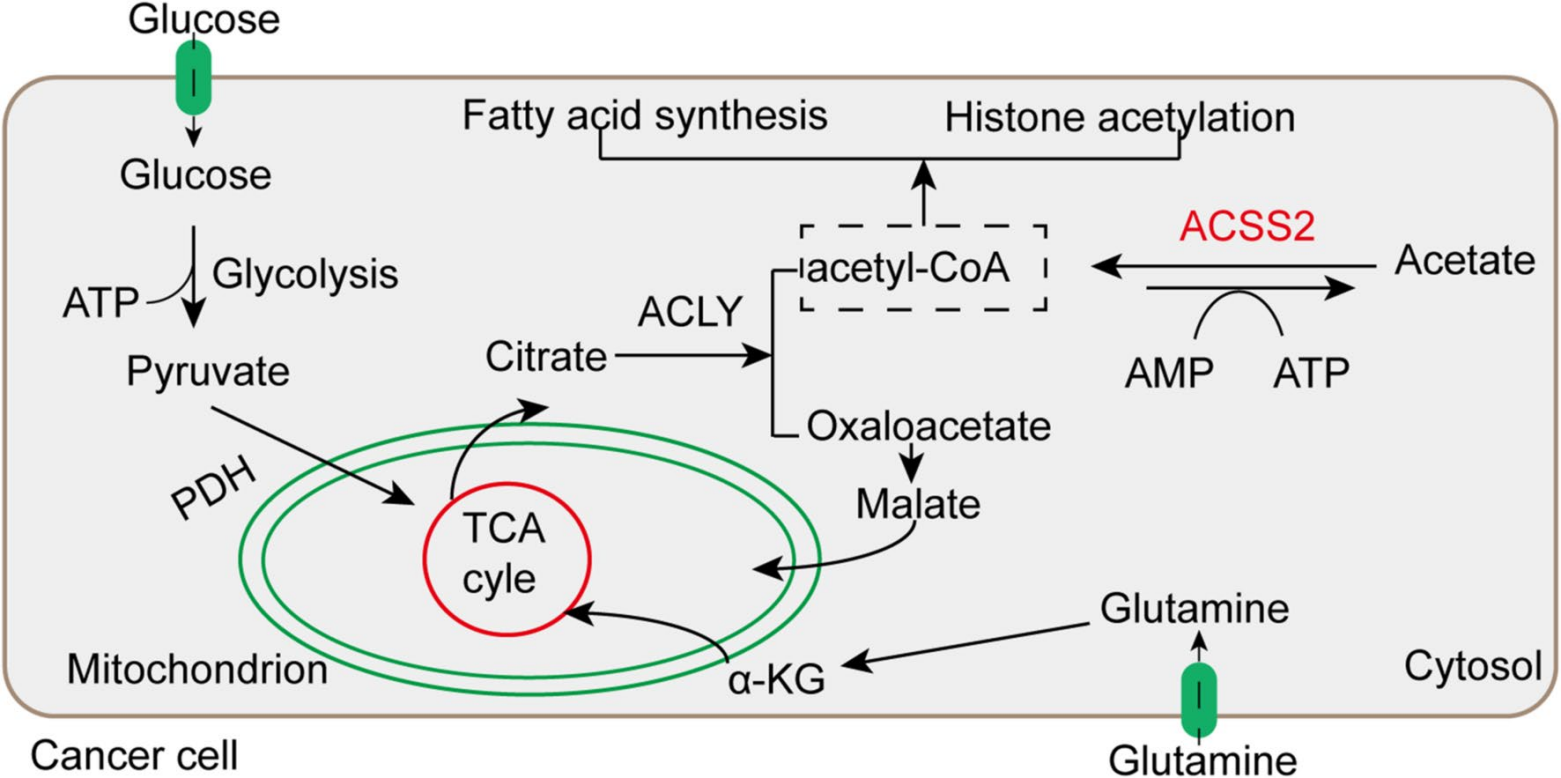
Synthesis and function of acetyl-CoA. Glucose is used to produce pyruvate through glycolysis, and the generated pyruvate is converted into acetyl-CoA in mitochondria and enters the tricarboxylic acid cycle. In this cycle, acetyl-CoA is converted to citrate, which is then transported to the cytoplasm and converted to acetyl-CoA and oxaloacetate. In turn, oxaloacetate is converted to malate under the regulation of ATP citrate lyase (ACLY), and malate is converted to oxaloacetate in mitochondria. Glutamine undergoes reductive metabolism to citrate to produce acetyl-CoA. ACSS2 catalyzes a reversible reaction between acetyl-CoA and acetate in cancer cells. Acetyl-CoA is used for lipid synthesis and histone acetylation

Tumor metabolic reprogramming is controlled primarily by proteins that are involved in programming the hallmarks of cancer [19, 20]. Acetyl-CoA, as an energy substrate, has a crucial function in the tricarboxylic acid cycle and serves as a primary substrate for fatty acid synthesis, cholesterol synthesis and histone acetylation [21–23]. As shown in Fig. 2, the biosynthesis of fatty acids in the cytoplasm of tumor and nontumor cells, acetyl-CoA is the initiating material in this process. Acetyl-CoA is the initiating substrate in fatty acid biosynthesis in the cytoplasm of both tumor cells and nontumor cells. In mitochondria, acetyl-CoA is usually generated from pyruvate. Citric acid is cleaved in a cytoplasmic reaction catalyzed by citrate lyase to form acetyl-CoA [24–26]. Under physiological conditions, the synthesis of citrate and its transport to the cytoplasm are dependent on the metabolic conditions and the tissue. Pyruvate is produced from glucose through glycolysis and enters mitochondria, where, through a reaction catalyzed by the pyruvate dehydrogenase complex, it is used to produce NADH, CO_2_ and acetyl-CoA. This acetyl-CoA then enters the tricarboxylic acid cycle. First, in mitochondria, citric acid is produced from acetyl-CoA and oxaloacetate via a condensation reaction. Citric acid is not readily oxidized. However, by aconitase dehydration and rehydration reactions, generating isocitrate. Isocitrate is easily oxidatively decarboxylated to α-ketoglutarate by isocitrate dehydrogenase, which in turn undergoes oxidative decarboxylation to generate succinyl-CoA. Succinyl-CoA is then hydrolyzed in a reaction catalyzed by succinyl-CoA synthetase, and a phosphate group is transferred to GDP to generate GTP and succinate. Succinic acid then undergoes dehydrogenation to form fumarate under the catalytic action of succinate dehydrogenase. Fumarate is hydrated to produce malate under the catalytic action of fumarate dehydrogenase. Malate is converted into oxaloacetate by dehydrogenation catalyzed by malate dehydrogenase, and the regenerated oxaloacetate can reenter the tricarboxylic acid cycle for continued synthesis of citric acid. In addition, the generated citric acid can be transported to the cytoplasm via tricarboxylic acid carriers on the intima. Via the catalytic action of citrate lyase and the consumption of adenosine triphosphate (ATP), citric acid is cleaved into oxaloacetate and acetyl-CoA. Under the catalytic action of malate dehydrogenase, oxaloacetate is reduced to malate. Malate is recycled to pyruvate under the catalytic action of malic enzyme, and pyruvate is transported back to mitochondria via mitochondrial carriers and is carboxylated to oxaloacetate. This new oxaloacetate can undergo a condensation reaction with acetyl-CoA to form citric acid, which begins the next cycle [27]. AKT is activated by insulin in adipose tissue, and activated AKT mediates the phosphorylation of ATP citrate lyase (ACLY) at residue 454, activating ACLY and promoting acetyl-CoA production from citrate [28]. Therefore, acetyl-CoA is crucial to the survival of cancer cells [29, 30]. Acetyl-CoA in tumors is produced mainly by decomposition of glucose from pyruvate to citric acid in the tricarboxylic acid cycle. However, accumulating data suggest that cancer cells can also produce acetyl-CoA by reductive metabolism of glutamine into citrate [31–33]. In addition, another route to generate acetyl-CoA in tumor cells is via ACSS, using acetate, coenzyme A, ATP, etc., to generate acetyl-CoA [11, 34]. ACSS is thought to be crucial in acetate/CoA metabolism in tumor cells [35]. ACSS1, ACSS3 and cytoplasmic ACSS2 play a major role in acetate metabolism [11, 36]. ACSS2 is mainly responsible for both lipid synthesis and histone acetylation [37, 38]. Through an assay of radiolabeled acetate uptake, a study showed that acetate uptake was increased in cancer cells compared with normal cells, and ACSS2 was found to be involved in the process of acetyl-CoA synthesis from acetate [35]. Furthermore, ACSS2-mediated acetate uptake is related to de novo synthesis of fatty acids in cancer cells. More importantly, acetyl-CoA converted from acetate is thought to be beneficial to tumor cells, especially when cellular glucose uptake is low, limiting regeneration of acetyl-CoA from citrate [39]. ACSS is generally considered to be involved in the conversion of acetic acid to acetyl-CoA through a forward reaction [8]. Interestingly, recent evidence from in vitro studies on various tumor cell lines has shown that ACSS2 also catalyzes a reverse reaction converting acetyl-CoA to acetate in cancer cells [39]. In tumor cells, this reverse reaction catalyzed by ACSS2 produces less ATP than the forward reaction and thus serves mainly to maintain acetyl-CoA homeostasis in tumor cells. In conclusion, ACSS2 mediates the reversible conversion of acetyl-CoA and acetate in cancer cells, which is key to understanding the survival of cancer cells.

## ACSS2 and lipid metabolism

ACSS2 has the dual properties of functioning as an adipogenic enzyme and in regulating the stress response and performs these different functions according to the nutritional status and stress conditions [18, 40, 41]. Under normal conditions in the absence of injury or stress, ACSS2 acts mainly as a cytoplasmic lipogenic enzyme, promoting the synthesis and storage of lipids. On the other hand, under nutrient deprivation, stress or injury, ACSS2 instead acts as a regulator to induce fatty acid oxidation and autophagy, thereby maintaining energy homeostasis. The expression of ACSS2 can thereby be activated by sterol regulatory element-binding proteins (SREBPs), which are targeted by the transcription factor Specificity protein 1 (Sp1), promoting ACSS2 expression through a reduction in fatty acid levels [42]. Data from other mouse studies also support the role of ACSS2 in lipid metabolism. ACSS2 expression in mouse liver and white adipose tissue was found to be downregulated under abstinence and increased under feeding in a manner dependent on sterol regulatory element-binding protein 1 (SREBP-1). Insulin positively regulates the expression of ACSS2, and a reduction in insulin levels reduces the expression of ACSS2 [43]. Deletion of ACSS2 in mice does not cause significant phenotypic differences, and these mice are fertile and produce normal litters. However, in ACSS2 knockout mice fed a high-fat diet, less fat was distributed in adipose tissue, and the expression of transcription factors regulating cholesterol and unsaturated fatty acid synthesis was decreased [8, 43, 44]. When cellular cholesterol levels fall below the threshold, proteases begin to act on SREBPs, and the active protein fragment produced by nuclear-translocated proteases binds to sterol regulatory elements (SREs) and promotes the expression of genes related to cholesterol and fatty acid synthesis. ACSS2 expression can be promoted by low cholesterol and fatty acid concentrations and inhibited by high cholesterol and fatty acid concentrations. The ACSS2 gene promoter contains 8 SREs and also contains Sp1 and Sp3 binding sites. As determined in wild-type mice, SREBP cooperates with the transcription factor liver X receptor, accentuating the importance of ACSS2 in lipid synthesis [45]. When mice rapidly ingest a large amount of fructose, which cannot be fully absorbed by the small intestine, the gut microbes metabolize fructose into short-chain fatty acids such as acetate and deliver it to the portal vein. This acetate is then used to produce acetyl-CoA via hepatic ACSS2, promoting fatty acid synthesis and fostering the formation of fatty liver [46]. Acetate not only promotes adipogenesis through ACSS2 but also modulates metabolic pathways by altering the histone arrangement and acetylation [47, 48]. In addition, in fasted knockout mice, glucose and ketone body levels in serum were decreased and serum levels of nonesterified fatty acids were increased [45]. Indeed, ACSS2 knockout has an extensive influence on the uptake, metabolism, and utilization of tissue-specific nutrients. Most of these studies were performed on hepatic tissue, adipose tissue, or cultured cells. However, ACSS2 may play a role other than being involved in lipid metabolism in other tissues.

## ACSS2 and hypoxia

**Fig. 3:**
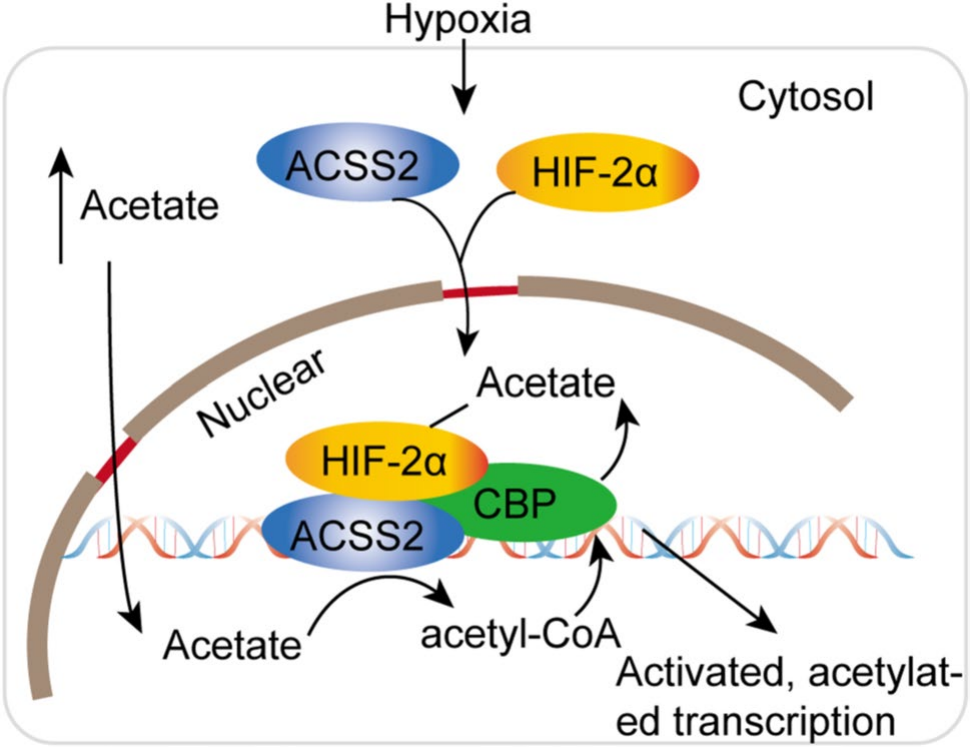
ACSS2 utilizes acetate to activate and acetylate transcription factors under hypoxic conditions. Under hypoxic conditions, acetate, ACSS2, and the transcription factor HIF enter the nucleus. In the nucleus, the acetate concentration increases, and HIF-2α is acetylated and recruited to form the CBP/HIF-2α complex. This complex activates transcription and acetylation

Hypoxia is usually correlated with the tumor microenvironment (TME), causing the activation of the transcription factor hypoxia-inducible factor (HIF) in tumor tissues [49, 50]. However, the HIF transcriptional control system is very complex and can regulate multiple signaling pathways, such as the adenosine 5'-monophosphate-activated protein kinase (AMPK) signaling pathway [51] and Notch signaling pathway [52]. Under hypoxic conditions, HIF-2α acetylation was found to be a crucial regulator of erythropoietin (EPO) expression [53]. Erythropoietin is synthesized in the kidney or liver in adult mammals, and this process orchestrates erythropoiesis and is modulated by the stress response transcription factor hypoxia-inducible factor-2 (HIF-2). Acetylation of HIF-2α by the lysine acetyltransferase CREB-binding protein (CBP) is crucial to efficient HIF-2-dependent induction of erythropoietin expression under hypoxic conditions. Studies have further shown that in erythropoietin-producing liver tumor cells or organs in mice in hypoxia or with severe anemia, the elevated acetate level requires ACSS2 to bind CBP acetylation to the formation of the CBP-HIF-2α complex, which is recruited to the erythropoietin enhancer while efficiently inducing erythropoietin gene expression. Moreover, the ACSS2/HIF-2 signaling pathway is activated under both hypoxic and glucose deprivation conditions. As shown in Fig. 3, under stress conditions, acetate concentrations increase as HIF-2α is acetylated and recruited to form the CBP/HIF-2α complex. Exogenous acetate further promotes HIF-2α acetylation, CBP/HIF-2α complex formation, and HIF-2 signaling, while tumor growth and metastasis in mice also increase in an ACSS2- and HIF-2-dependent manner [54]. Similarly, in glucose-deprived or hypoxic tumor cells, the intracellular acetate level and the nuclear translocation of ACSS2 from the cytoplasm are increased. In the nucleus, HIF-2α is acetylated via ACSS2, with CPB as a coactivator, resulting in changes in histone acetylation and upregulation of transcription [55]. These studies highlight the function of transcription factor acetylation in regulating erythropoietin gene expression under the pathophysiological conditions of tissue hypoxia and the mechanism by which ACSS2 is involved in mediating this response. ACSS2 expression is upregulated in hypoxic states, and inhibition of ACSS2 in cancer cells during chronic hypoxia enhances cell death, suggesting that ACSS2 might function as a crucial factor in the survival of cancer cells under hypoxic conditions.

## Effects of ACSS2 on the AMPK signaling pathway and autophagy

**Fig. 4:**
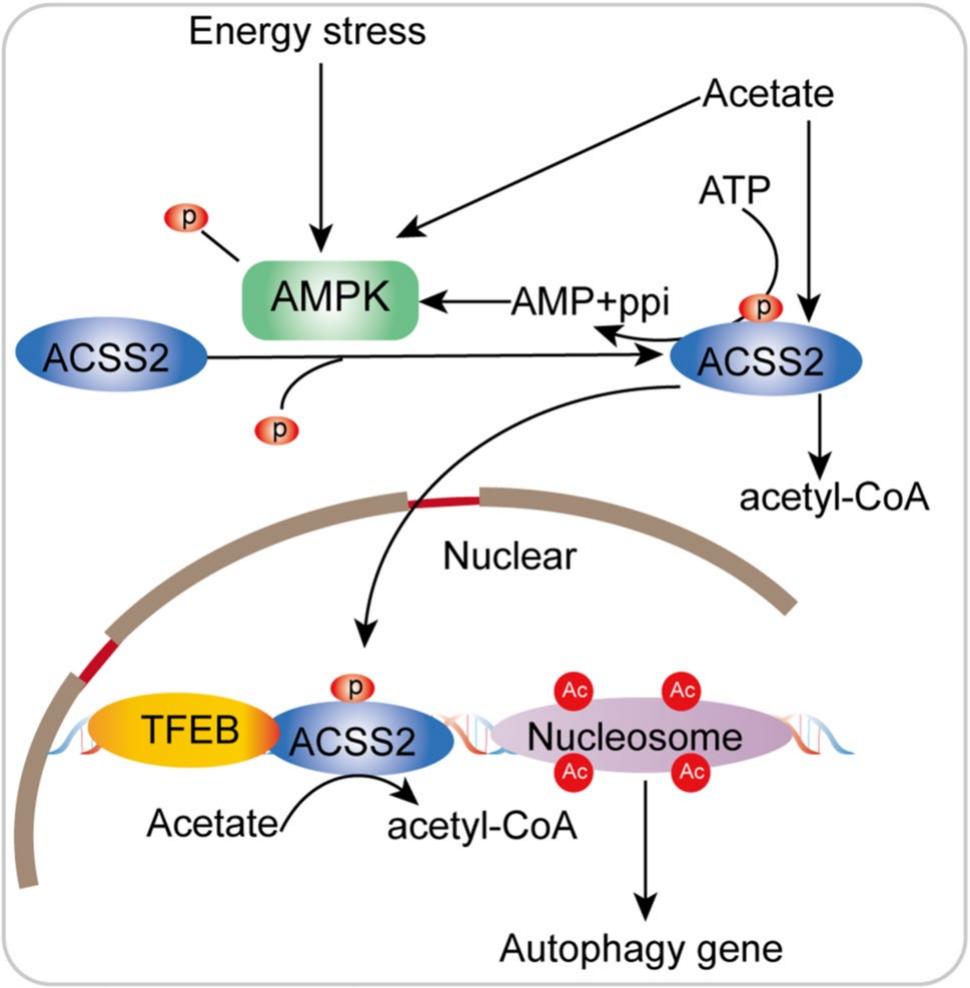
Interaction between ACSS2 and AMPK. Energy stress and acetate promote the phosphorylation and activation of AMPK. AMPK phosphorylates ACSS2 at S659 and leads to its nuclear translocation. Nuclear ACSS2 and TFEB form a complex that additionally acetylates histones through the production of acetyl-CoA. ACSS2 drives AMPK activation by converting acetate and ATP to acetyl-CoA and AMP

An ATPase is required for the conversion of acetate and CoA to acetyl-CoA and adenosine monophosphate (AMP), a metabolic pathway that can function in anabolic and catabolic processes. Indeed, evidence indicates that AMPK can act as a regulator of the function and subcellular localization of ACSS2 [56]. AMPK is generally considered the major regulatory enzyme controlling central energy metabolism [57]. AMPK, a serine/threonine kinase, is a main regulator of energy metabolites that downregulate the cellular energy state by catabolic stimulation and energy derivation, while preventing biosynthetic pathways from compensating for the cellular energy supply [58, 59]. Therefore, the nutritional status and energy expenditure level regulate AMPK activity [60]. In this context, AMPK can regulate key enzymes involved in many main metabolic pathways through phosphorylation [61]. Activation of AMPK for nutrient deprivation and energy expenditure involves multiple mechanisms, including activation of upstream kinase phosphorylation [62, 63], as well as activation at low ATP levels and high AMP and adenosine diphosphate (ADP) levels [64]. The specific response of AMPK to different stimuli is determined by heterodimerization of the isomers and raises the question about the function of acetate in regulating AMPK and metabolic processes [65]. Acetate promotes the phosphorylation and activation of AMPK. Acetate directly activates AMPK by reducing the level of intracellular ATP along with inducing an increase in the AMP level and the AMP/ATP ratio, which is followed by dose-dependent phosphorylation and increased activation of AMPK. Researchers have found evidence suggesting that acetate-mediated activation of the AMPK signaling pathway can promote lipid oxidation and reduce lipid synthesis [66, 67]. However, ACSS2 can promote adipogenesis, and AMPK may antagonize this effect. Acetate promotes energy production or lipid synthesis, requiring the conversion of cytoplasmic or nuclear ACSS2 to acetyl-CoA, subsequently activating the AMPK pathway to replenish cellular energy. More recently, studies have begun to elucidate the connection between ACSS2 and AMPK, leading to a link to autophagy. Phosphorylation of the serine (Ser, S) 659 of ACSS2 by AMPK exposes a nuclear localization signal on ACSS2 and leads to its translocation into the nucleus. Nuclear ACSS2 and transcription factor EB (TFEB) form a complex, that deacetylates histones to generate acetate locally, while producing acetyl-CoA to upregulate histone acetylation in the promoter regions of TFEB target genes [41]. Based on the available evidence, as shown in Fig. 4, we believe that the signaling pathways connecting ACSS2 and AMPK are bidirectional and that a positive feedback loop may be formed under certain physiological contexts. However, in response to abstinence or cellular stress (such as hypoxia), AMPK redirects ACSS2 from an adipogenic function to a regulatory function. ACSS2 catalyzes the production of AMP, which in turn signals a decline in cellular energy reserves. ACSS2 drives protein acetylation and AMPK activation by converting acetate and ATP into acetyl-CoA and AMP. After phosphorylation by AMPK, ACSS2 promotes the formation of acetyl-CoA while reducing the ATP level and increasing the AMP level (Fig. 4). Acetyl-CoA deficiency causes autophagy, while a surplus of acetyl-CoA represses autophagy [68]. ACSS2 regulates autophagy in a nutrient-sensitive manner through acetyl-CoA production and consequent protein acetylation [40]. Generally, high levels of acetyl-CoA promote protein acetylation via acetyltransferases, causing an overall increase in protein acetylation. In proteins associated with autophagic responses, hyperacetylation is followed by fast and immediate posttranslational inactivation over a longer time period through nutrient-sensitive regulatory kinase pathways and epigenetic regulation through the expression of autophagy-related genes [69]. Since ACSS2 causes an increase in acetyl-CoA and contributes to acetate levels, an increase in intracellular acetate results in an ACSS2-mediated increase in acetyl-CoA, which subsequently induced protein acetylation through acetyltransferases. Inhibition of cellular ACSS2 leads to a decline in acetyl-CoA levels, subsequently causing a decrease in cytoplasmic protein acetylation levels and, ultimately, an increase in autophagy [68]. Via this mechanism, protein acetylation can function as a crucial sensor of nutritional status and is associated with autophagy by modulation of protein acetylation levels, while ACSS2 catalyzes the production of some of the acetyl-CoA required for these protein acetylation reactions.

## ACSS2 and tumors

**Fig. 5:**
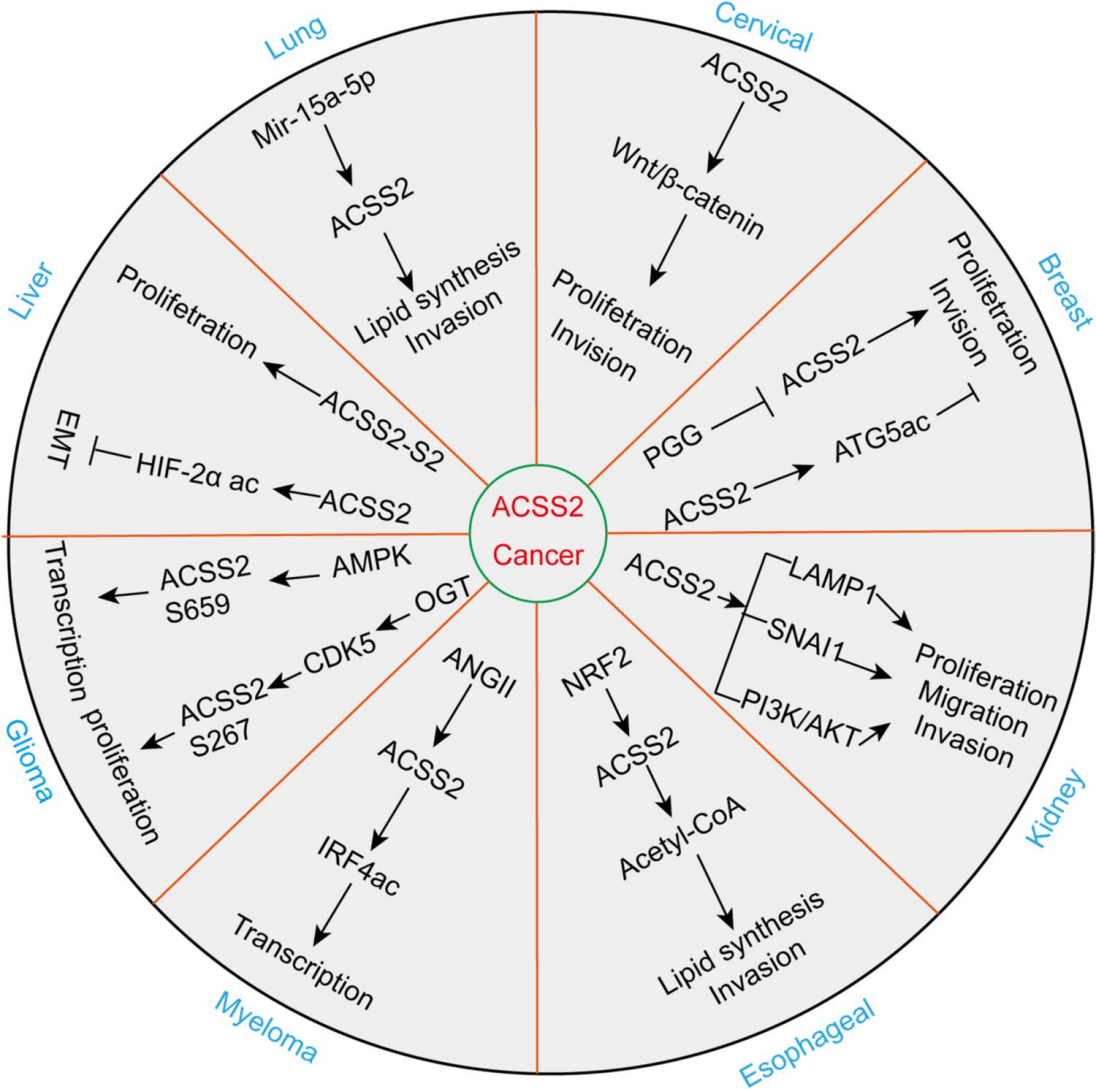
Regulatory roles of ACSS2 in various tumors

ACSS2 is considered a target for tumor therapy and serves an important function in cell proliferation, gene expression regulation, and bioenergetics [70, 71]. In most cell types, ACSS2 can be found in the cytoplasm and nucleus. However, the nuclear expression of ACSS2 is much higher than the cytoplasmic expression of ACSS2 in the cerebrum of the adult rat [72]. An important role of ACSS2 is the nuclear production of acetyl-CoA to achieve the acetylation of histones and transcription factors [73]. The important nuclear localization of ACSS2 in most cell types is ignored, partially due to the role of ACSS2 in cytoplasmic lipid synthesis. ACSS2 has been found to primarily undergo translocation from the cytoplasm to the nucleus [55]. ACSS2 translocation is tightly connected with hormonal and transcription factor signaling from the cell periphery to the nucleus to generate or promote the transcription of specific genes. Clearly, ACSS2 functions as a regulator that affects gene transcription in many ways, as other transcription factor complex enzymes can be assembled; however, ACSS2 is also the only enzyme known to utilize free acetate inside the nucleus, which allows it to produce local acetyl-CoA if the local acetate level in the nucleus increases [18, 73]. Therefore, ACSS2 facilitates all nuclear acetyltransferase activity. ACSS2 promotes nuclear acetylation reactions through localized production of acetyl-CoA. Therefore, ACSS2 can affect gene transcription by controlling two crucial acetylation reactions (histone- and transcription factor-specific acetylation). By promoting the acetylation of histones and transcription factors, ACSS2 influences metabolic reprogramming and cell cycle progression in tumor cells (Fig. 5).

### Liver cancer

In human hepatocellular carcinoma, tumor subtypes can be stratified according to acetate metabolism. ACSS1 is associated with the iHCC3 subtype, with the lowest survival rate; ACSS2 is more closely related to iHCC2, with an intermediate survival rate; and ACSS3 is related to iHCC1, the subtype with the longest patient survival time [74]. Recently, two alternative transcription start sites in the ACSS2 gene were found in hepatocellular carcinoma cells, which might lead to clarification of the dual roles of ACSS2 in facilitating and suppressing cancer invasiveness [75]. These alternative transcription start sites lead to the generation of two distinct ACSS2 transcript isoforms, ACSS2-S1 and ACSS2-S2. These isoforms of ACSS2 have distinct subcellular localization characteristics and roles. Expression of the ACSS2-S1 isoform is mainly cytoplasmic and is less common in liver tumor tissues than in normal tissues. The ACSS2-S2 isoform is expressed in both the nucleus and cytoplasm, and its expression is greatly increased in liver cancer tissues. In vitro experiments have shown that overexpression of ACSS2-S2 can enhance the proliferation and invasion abilities of tumor cells, while overexpression of ACSS2-S1 made no obvious difference. Paradoxically, also in HCC studies, ACSS2 knockdown was shown to cause HIF-2α deacetylation, which subsequently increases cell migration and invasion and induces epithelial–mesenchymal transition, indicating poor prognosis [76]. Furthermore, hepatocellular carcinoma upregulates the expression of ACSS2 to utilize acetate [38]. Under normoxic culture conditions, hepatocellular carcinoma cells synthesize acetyl-CoA, which is transported to mitochondria as an essential energy source; in contrast, under hypoxic culture conditions, the expression of ACSS2 increases nearly fivefold [18]. In addition, ACSS2 was shown to reduce tumor burden in a liver cancer model [45]. Acetate increases the acetylation levels of H3K9, H3K27 and H3K56 in the fatty acid synthase (FASN) and acetyl-CoA carboxylase alpha (ACACA) promoter regions in HepG2 cells under hypoxic conditions to promote lipid synthesis. Simultaneous knockdown of ACSS1/2 significantly inhibits acetate-induced increases in the acetylation levels of H3K9, H3K27, and H3K56 and reduces the partial contribution of acetate to palmitate and stearate synthesis, as well as acetate-induced cell survival. In addition, immunohistochemical (IHC) analysis revealed that the expression of ACSS1/2 was positively correlated with the H3 acetylation and FASN expression in human HCC [77]. These findings show that ACSS2 activity contributes to the initiation and development of liver cancer.

### Glioma

Glioblastoma is the most prevalent adult primary brain tumor. Glioblastoma cells can oxidize both glucose and acetate but not glutamine [78]. Since acetate prevents the division of glioblastoma-like stem cells, compounds that induce an increase in circulating acetate levels have been suggested for use in treating glioblastoma [79]. Recent data suggest that ACSS2 levels are moderate in neurons and oligodendrocytes and that ACSS2 is localized mainly in the nucleus [13]. During serum deprivation, ACSS2 translocates from the cytoplasm to the nucleus, which is required for neural differentiation [73]. After differentiation, ACSS2 binds to chromatin occupied by CBP, leading to the acetylation of H3K9, H3K27, and H4K5. Moreover, H3K9, H3K27, H4K5, and H4K12 undergo an even higher level of acetylation. ACSS2 provides acetyl-CoA for CBP in this process. Further studies showed that glucose restriction leads to AMPK-mediated phosphorylation of ACSS2at S659, exposes a nuclear localization signal for importin A5 binding and nuclear translocation. Nuclear ACSS2 interacts with TFEB and binds to the promoter region of autophagy genes; ACSS2 binds to acetate generated via histone deacetylase activity to produce acetyl-CoA for histone H3 acetylation catalyzed by acetyltransferases, thereby promoting lysosomal biogenesis, autophagy and brain tumorigenesis [40]. Recent studies have shown that glioblastoma cells preferentially convert acetate to acetyl-CoA as an energy source to support tumor growth. O-GlcNAc transferase (OGT) regulates the phosphorylation of ACSS2 at S267 through cyclin-dependent kinase 5 (CDK5), which in turn mediates acetate-dependent acetyl-CoA and lipid production. In mouse experiments, hyperphosphorylation of ACSS2 at S267 was found to promote tumor growth. Therefore, modulating the OGT/CDK5/ACSS2 axis constitutes a new strategy for tumor-targeted therapy [80].

### Lung cancer

In non-small-cell lung cancer, the expression levels of ACSS2 phosphorylated at S659(ACSS2-pS659) in 303 surgical specimens were analyzed by immunohistochemistry. Acss2 phosphorylation at S659 was greatly increased in tumor tissues compared with the surrounding normal tissues and stimulated the nuclear translocation of ACSS2, which is considered a reliable prognostic marker for poor survival. The increase in phosphorylation of ACSS2 at S659 also promoted tumor growth [40, 81]. Abnormal lipid metabolism could facilitate the progression and metastasis of lung cancer [82]. miR-15a-5p was found to greatly inhibit fatty acid synthesis in lung tumor cells by suppressing acetate uptake. In addition, in lung tumor cells, nuclear localization of ACSS2 occurs under hypoxic conditions, and miR-15a-5p is translocated into the nucleus to bind to the 3'-UTR of ACSS2, which inactivates the expression of ACSS2, reduces acetate uptake and inactivates acetyl-CoA production, thereby reducing histone H4 acetylation. Finally, miR-15a-5p was found to suppress lung cancer cell metastasis by inhibiting ACSS2-mediated lipid metabolism [83].

### Breast cancer

The antitumor activity of the polyphenol polyclonal gamma globulin (PGG), isolated from eucalyptus leaves, has been identified. Several genes strongly related to pyruvate metabolism, glycolysis/gluconeogenesis, and tyrosine metabolism, including ACSS2, were found to be significantly downregulated in PGG-treated MDA-MB-231 breast cancer cells. This finding indicated that ACSS2 might be a metabolic target gene in breast cancer [84]. In human breast tumors, the ACSS2 copy number is increased, promoting breast cancer cell growth under hypoxic and low-fat conditions through acetate uptake. Under stress, downregulation of ACSS2 expression was found to reduce the growth of xenograft tumors, suggesting that ACSS2 plays an important role in tumor cell survival and growth in the harsh breast cancer microenvironment [18, 85]. It Moreover, overexpression of ACSS2 was found to increase the H3K27 acetylation (H3K27ac) in the promoter region of ATG5 and to maintain autophagic flux while reducing the proliferation, migration and invasion of breast cancer cells [86]. Recent studies have reported that 4-hydroxytamoxifen (4-OHT) induces ACSS1 and ACSS2 mRNA and protein expression in estrogen receptor-α-positive (ER^+^) breast cancer cells and derived 4-OHT-resistant cells to increase their survival. Knockdown of ACSS1 and ACSS2 or treatment with an ACSS2-specific inhibitor was found to result in loss of cell viability and a reduced proliferative capacity. Furthermore, ACSS2 promotes increased cancer cell survival partially by maintaining autophagy [87]. Currently, the mechanism of action of ACSS2 in breast cancer remains unclear, and more studies are needed for a better understanding.

### Renal cell carcinoma

Initially, ACSS2 expression in renal cell carcinoma tissues was found to be markedly higher than that in adjacent tissues, and this increased expression was positively related to tumor metastasis. ACSS2 showed no influence on the proliferation and apoptosis of tumor cells, but reduced ACSS2 expression inhibited lysosome-associated membrane protein 1 (LAMP1) protein expression compared with that in the control group, thus preventing the migration and invasion of renal cell carcinoma cells [88]. Studies have also shown that high expression of ACSS2 is correlated with advanced T stage and lymph node metastasis; blockade of ACSS2 inhibits the growth, migration and invasion of renal cell carcinoma cells possibly by suppressing the PI3K/AKT signaling pathway, while overexpression of ACSS2 enhances these effects [89]. Researchers have found that acetate is associated with tumor metastasis [90]. Acetate was found to increase the expression of SNAI1 (a zinc finger transcriptional repressor) and ACSS2 in renal cancer cells under glucose-limiting conditions. ACSS2 knockdown greatly reduced acetate-induced SNAI1 expression and cell migration, while ACSS2 overexpression increased the SNAI1 level and facilitated H3K27 acetylation to promote cell migration [91]. These results strongly indicate that targeting ACSS2 is a potential therapeutic strategy for metastatic cancer.

### Gastrointestinal cancer

In colorectal cancer, the significance of ACSS2 expression may be different from that in other malignancies, since normal colon cells utilize short-chain fatty acids as a fuel source, unlike the mechanism of ACSS2, which utilizes acetic acid as a carbon source for tumor cells. Decreased ACSS2 expression in colorectal cancer is strongly related to advanced tumor–node–metastasis (TNM) stage, poor differentiation, and cancer recurrence, and is an independent prognostic factor for poor 5-year progression-free survival. Downregulation of ACSS2 expression is a metabolic marker of tumor progression and aggressive behavior in colorectal cancer [92]. Similarly, ACSS2 expression is low in gastric cancer, resulting in poor survival and prognosis [93]. However, in a study of esophageal squamous cancer cells, ACSS2 expression was promoted in tumor cells, although these cells were less responsive to nutrient deprivation than were normal cells. In addition, siRNA transfection significantly inhibited ACSS2, resulting in decreased proliferation of cancer cells under nutritional stress, suggesting that acetate utilization by ACSS2 is a crucial factor in these cells [94]. In another study, overactive nuclear factor erythroid 2-related factor 2 (NRF2) upregulated ACSS2. In esophageal cancer cells and the mouse esophagus, knockout of either NRF2 or ACSS2 reduced ACSS2 expression, which subsequently decreased acetyl-CoA and ATP levels. Moreover, lipid synthesis in esophageal carcinoma cells was decreased, and the invasion ability of these cells was weakened [95].

### Myeloma

Overexpression of ACSS2 can be identified in myeloma cells from obese patients and promotes myeloma progression. Adipocyte-derived angiotensin II stimulated ACSS2 expression in myeloma cells, while upregulated ACSS2 interacted with the oncoprotein interferon regulatory factor 4 (IRF4) to enhance the its stability and expression by mediating its acetylation. Target gene transcription promoted tumorigenesis, and inhibition of the angiotensin II/ACSS2 axis inhibited the progression of obesity-associated myeloma [96]. In glucose-deprived melanoma cells, acetate or glutamine alone was not sufficient to maintain cell viability, while the combination of acetate and glutamine significantly restored cell viability. Acetate addition significantly enhanced the expression of ACSS1 and ACSS2. Furthermore, knockdown of either ACSS1 or ACSS2 significantly reduced tumor growth in mice [97].

### Other tumors

ACSS2 expression in cervical tumor tissue was found to be notably higher than that in adjacent normal tissue. Silencing ACSS2 expression may inhibit nutrient deprivation-induced migration and invasion of cervical cancer cells by suppressing Wnt/β-catenin signaling pathway activity [98]. In cervical squamous cell carcinoma (CESC), ACSS2 expression is associated with infiltration of B cells, CD4^+^ and CD8^+^ T cells, and cancer-associated fibroblasts (CAFs), and high ACSS2 expression is associated with shorter overall survival times. ACSS2 is thus a potential diagnostic and prognostic biomarker for CESC [17]. Cisplatin is a crucial chemotherapeutic agent in the treatment of metastatic bladder cancer. However, drug resistance often limits its use. Recently, altered lipid metabolism was found in bladder cancer, and ACSS2 expression was found to be higher in the tissues of patients with cisplatin-resistant bladder cancer. Further studies have shown that ACSS2 but not the established ACLY promotes tumor resistance to cisplatin by providing acetyl-CoA to cisplatin-resistant bladder cancer cells via glucose-derived endogenous acetate [99].

## Development of ACSS2 inhibitors

There are several clinically developed inhibitors of ACSS2. The developed ACSS2 inhibitor was able to reduce cell viability in the breast cancer cell line MDA-MB-468 as well as in subcutaneous xenografts in mice [100]. Moreover, amide-substituted condensed pyridine derivatives and novel substituted tetrazoles were found to inhibit ACSS2 [101, 102]. N-(2,3-Di-2-thienyl-6-quinoxalinyl)-N'-(2-methoxyethyl) urea is one of the most potent inhibitors of ACSS2 and can inhibit cellular [14C]-acetic acid uptake into lipids and histones [38]. VY-3–135 is a potent and selective ACSS2 inhibitor that inhibits ACSS2 activity both in vitro and in vivo and inhibits in vivo tumor growth in breast cancers with high ACSS2 expression [85, 103, 104]. MTB-9655, a small-molecule inhibitor of ACSS2, is a potential therapeutic agent for cancer patients and has entered a phase I (dose escalation) clinical trial (NCT04990739).

## Conclusion

Currently, tumor metabolism remains a popular research topic, and the discovery of the function of ACSS2 has opened up a new direction for tumor metabolism research. Research on ACSS2 in tumors is gradually increasing, and the identified effects of ACSS2 on the biological characteristics of tumor cells are not completely consistent. The molecular mechanistic role of ACSS2 in most tumors is to generate acetyl-CoA from acetate, which in turn participates in the acetylation of histones and genes, and promotes the synthesis of biological macromolecules such as lipids. ACSS2 is closely related to hypoxia due to the biological characteristics of tumor growth. In addition, under stress, the molecular signaling pathways in tumor cells undergo corresponding compensatory changes, and the compensatory increase in ACSS2 can maintain the survival of tumor cells. Research on the molecular mechanism by which ACSS2 mediates the occurrence and development of different tumors can provide us with new insights for understanding tumorigenesis and tumor development and identify new targets for molecular-targeted tumor therapy.
